# Talking about a Christine Borland sculpture: effective empathy in contemporary anatomy art (and an emerging counterpart in medical training?)

**DOI:** 10.1080/14702029.2015.1041743

**Published:** 2015-07-24

**Authors:** Craig Richardson, Christine Borland

**Affiliations:** ^a^ The College of Art, Design and the Built Environment, Nottingham Trent University, Burton Street, NottinghamNG1 4BU, UK; ^b^ Department of Arts, The University of Northumbria, BALTIC 39, 31–39 High Bridge, Newcastle upon TyneNE1 1EW, UK

**Keywords:** anatomy, medicine and contemporary art, sculpture, museum

## Abstract

This Introduction and interview discusses the poetical and empathic insights that are a key to the effectiveness of contemporary artist Christine Borland's practice and its relevance to the medical humanities, visual art research and medical students’ training. It takes place in a context of intensive interest in reciprocity and conversation as well as expert exchange between the fields of Medicine and Contemporary Arts. The interview develops an understanding of medical research and the application of its historical resources and contemporary practice-based research in contemporary art gallery exhibitions. Artists tend not to follow prescriptive programmes towards new historical knowledge, however, a desire to form productive relationships between history and contemporary art practice does reveal practical advantages. Borland's research also includes investigations in anatomy, medical practices and conservation.

## Introduction: Craig Richardson

**Figure 1.**
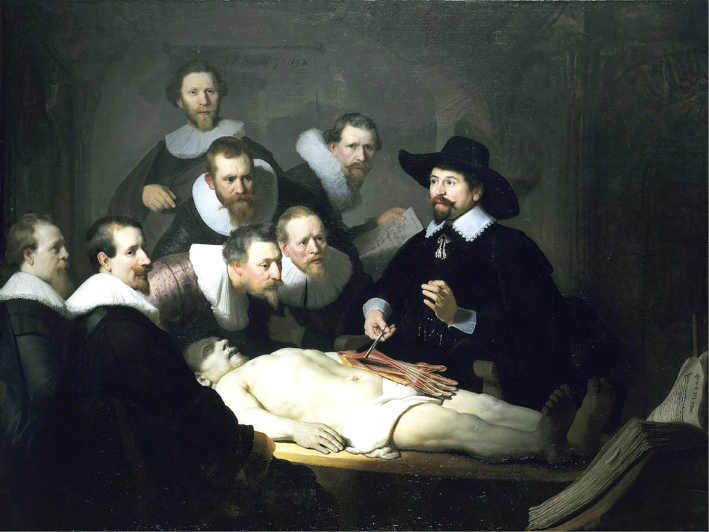
Rembrandt van Rijn, *The Anatomy Lesson of Dr. Nicolaes Tulp, 1632, (detail)*. Collection of Royal Picture Gallery Mauritshuis, The Hague.

Christine Borland's response to a mid-Victorian plaster cast, to which she devoted her attention between late 2010 and early 2012, can be contextualised through an incident in W.G. Sebald's meditation on transience *The Rings of Saturn* (1995). Sebald speculated the lesson represented underway in Rembrandt's *Anatomy Lesson* (1632) (Figure 1) had a punitive dimension and he highlighted how the inversion of the anatomical – one hand is pictured the wrong way round – at the painting's centre was intended to draw attention to the violence that has been doubly performed upon Kindt's executed body. It was:

with him, the victim, and not the Guild [of surgeons] that gave Rembrandt his commission, that the painter identifies. He alone sees that greenish annihilated body, and he alone sees the shadow in the half-open mouth and over the dead man's eyes. (1995 [English translation, 1998], 17)

Rembrandt's empathy is a reminder of the vital insights artists may bring to historical medical practices and their contemporary counterpart. Borland's encounter with the norms of specialised medical research often means a specifically empathic insight can be relocated into the space between their norms and then their historical or public presentation in galleries and Museums but also to feed back into contemporary clinical settings and training. In Borland's practice – to date – there is no direct engagement with patients in a clinical setting and the ethical frameworks for treatment would be prohibitive, however, her contingent dialogue with those in the medical and Museum professions means she often reaches an implicit point of convergence. Acting at one remove and as a generalist, her focus is on material culture and making a differently activated environment through a performative dimension; the culmination of her investigations (a more appropriate terms than research) involves new visualisations of old and new medical practices, while engaging differently and unconventionally with the technical apparatus and processes and/or overlooked ephemera found in these settings. In subsequent exhibitions the sometimes overlooked, incidental, ephemeral and technical elements are extracted from their context; such inversions and elisions enable another type of ‘knowing’. (Kohn 2011, 3–4).^1^

Her ‘conversational expert dialogue’, an informal method deployed by Borland during her initial investigations in medical environments, sometimes entails emergent surprises and discoveries that are often re-evaluated and synthesised for exhibition purposes. Borland produces art-works in which medical methodologies are not necessarily included within their exhibition, although such extrapolations are sometimes disseminated in accompanying catalogues and educational publications.

The interview's focal points are the decisions Borland made during a Production Residency and Exhibition at Glasgow Sculpture Studios in 2010–2011. We note how Borland's programme of plaster cast research was complemented by her previous exhibition *SimBodies* & *NoBodies* (2009) (Figures 3 and 4) at The Ormeau Baths Gallery, Belfast, which included sculptures derived from mannequins normally deployed in medical teaching to assist demonstrations of the Heimlich manoeuvre or contemporary prosthetic mannequins such as ‘Choking Charlie’.

**Figure 2.**
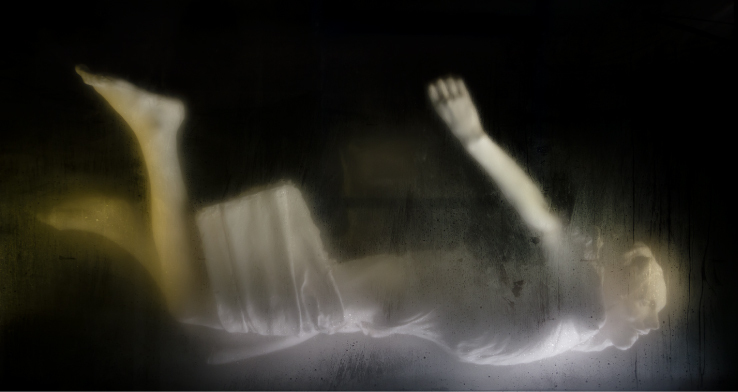
Christine Borland, Film Still – NoBodies; Cast From Nature, 2011, 12minute 30 second loop, H.D. Video, projected. (Made possible by The Morton Award for lens based media, 2010)

Considering both the 2011 plaster work and earlier mannequin projects we ask how an artist can raise questions and explore the ways in which the subject of scientific study can evoke empathy, through artistic interpretation of medical apparatus and anatomical representation. Such questions are discussed as creative and pedagogical issues. Her research for this stemmed from an initial encounter with a fibreglass cast permanently displayed in the Royal College of Surgeons (RCS) in Edinburgh. RCS Surgeons’ Hall Museum for the History of Surgery attributes the fibreglass as *Cast From Nature* and dated 1845, by [Sir] John Goodsir (1814–1867), and it is likely the fibreglass cast was taken from a plaster cast original in the nearby Anatomical Museum in Edinburgh University. There the body of a partially flayed man is posed after Michelangelo's Vatican *Pièta* (1499). The Glasgow Production Residency phase later culminated in the exhibition *Cast From Nature* at Glasgow Sculpture Studio a few days after this interview, and which restaged the recovery and representation of the plaster cast found in the basement of the Edinburgh Anatomical Museum. The installation was further synthesised at a second venue, Camden Arts Centre, May–July 2011, and elements have continued to appear in exhibitions at Edinburgh,^2^ Orkney and Liverpool (Figure 2).

**Figure 3.**
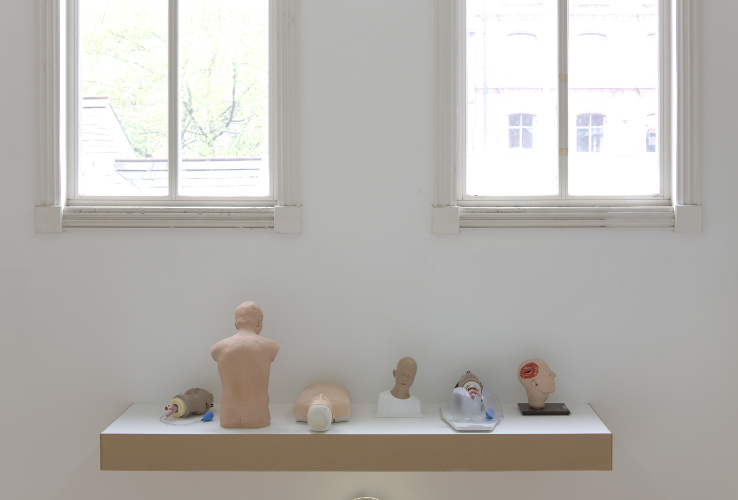
Christine Borland, SimBodies & Nobodies, 2009, Detail, Medical Manikins.

From the beginning of the Glasgow Sculpture Studios exhibition (late 2010) to the exhibition at Camden Arts Centre in May 2011 Borland seemed perplexed by her fascination with the plaster cast and sought to apply this in exhibition arrangements that emphasised its metaphysical value. Rather than providing anecdotal cover, the historical context is also fructifying, or at least the relationship between art and history is consolidated in the manner by which exhibition seemed to contain elements of conservation practice. Historical understanding has channelled Borland's empathic response but has not delimited its representation through, for instance, explanatory text panels.^3^ The materiality of plaster was very much in evidence in both exhibitions.^4^

**Figure 4.**
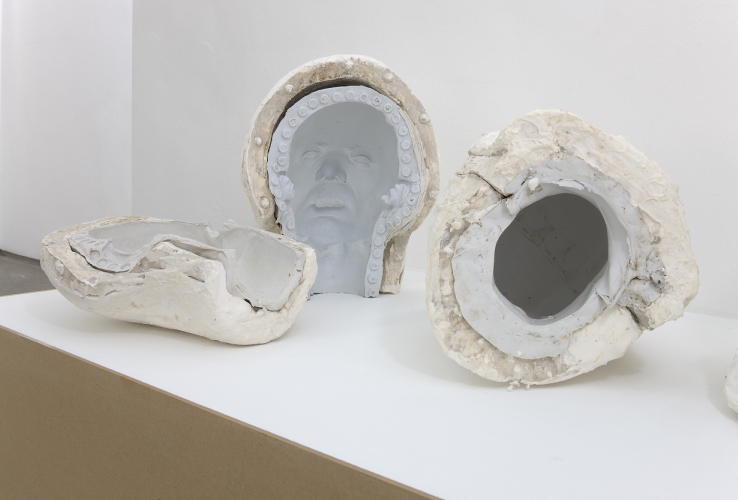
Christine Borland, SimBodies & NoBodies, 2009, Detail Plaster & Silicon Moulds.

**Figure 5.**
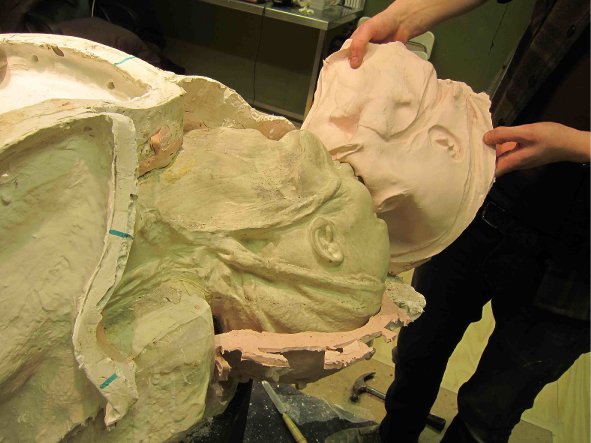
Christine Borland, Cast From Nature, Glasgow Sculpture Studios, 2010, Casting in Progress.

**Figure 6.**
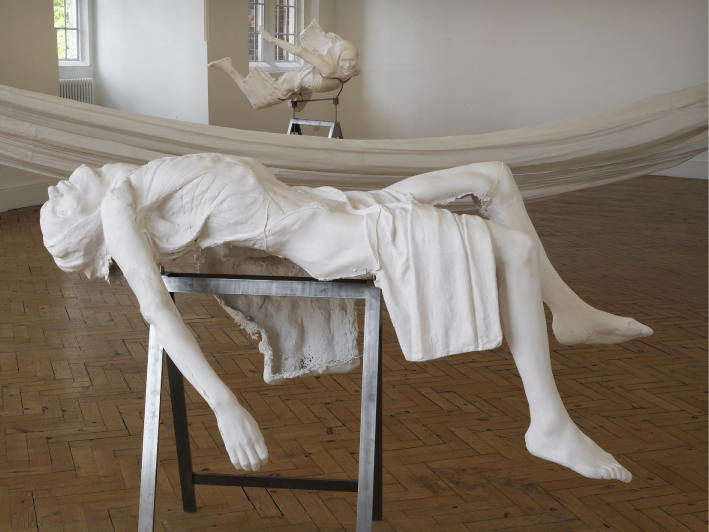
Christine Borland, Cast From Nature, 2011, Camden Arts Centre. (Photo: Andy Keate)

In Camden two plaster cast figures were separated by a long-draped plaster-soaked cloth diagonally traversing from opposite walls of the gallery that tended to emphasise horrific aspects of the two figures within a dramatic tableau (Figure 6). Once more the exhibition of these casts was an arena for subjective retrieval (a subjectivity Borland similarly describes in the progressive training of medical students studying anatomy in New Zealand). One of the casts, held in the classical Pieta recline but fixed upon a steel-rod rack, was positioned slightly higher than table height, coincidently Dr Tulp's pose when stationed in front of Rembrandt's famous group portrait. This steel-rod rack kept the cast figure's head in a painfully unsupported distortion with the throat pushed outwards, surely a choking position were the figure alive. Contradictions were evident. This Pieta derived plaster cast's eyes face the heavens, the pelvis clothed, the body given a semi-dignified appearance. Nevertheless the image is a body in an arc of pain. On the other side of the long brittle drape the same body cast was then displayed 180° facing downwards, the painful throat distension was now an unremarkable effect and the naturally dropping mouth was now seemingly alive and even responsive. The visage was placed at standing face height, the figure was no longer reclining, its flayed and drooping flesh now resembling a fast falling life rushing mercilessly downward towards oblivion. While this may have conjured the horror-fall of aerial victims the cast eyes no longer looked to the heavens but stared ahead with a languid but superior menace. Two figures, one classically reposed after death in contrast with the other, caught in the fast race towards death set in a room with an almost imperceptible even settling of fine chalk dust. Plaster is heavier than other dust motes.

**Figure 7.**
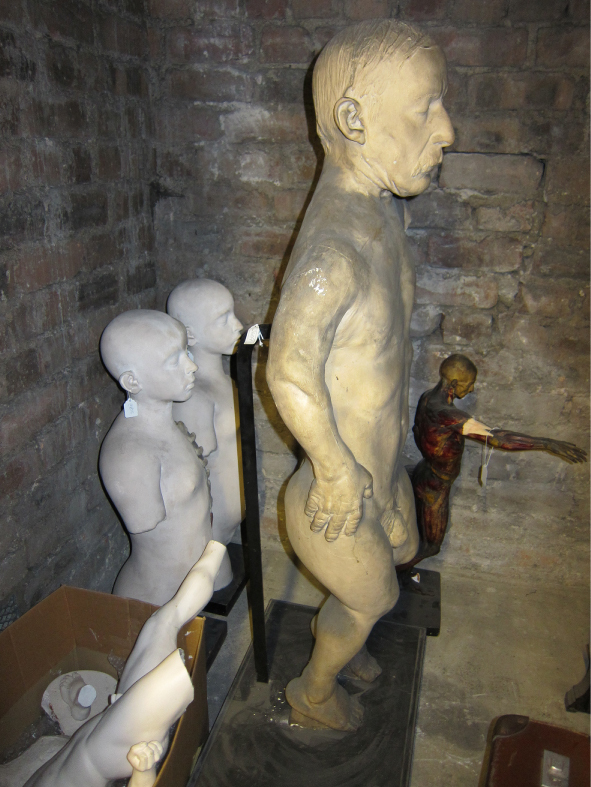
Basement, Edinburgh Anatomical Museum Stores.

**Figure 8.**
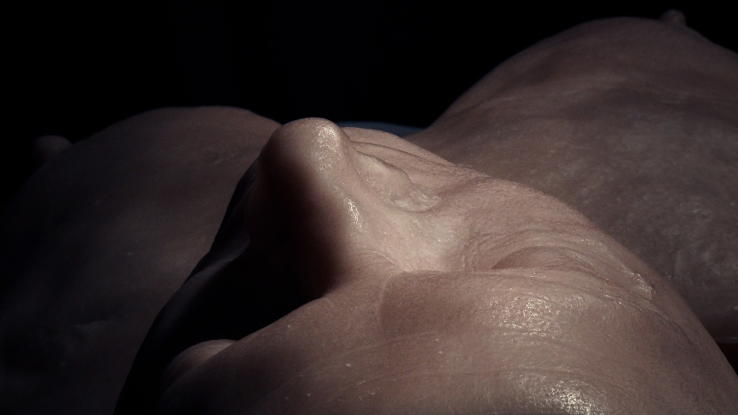
Christine Borland, SimWoman, 2010, Film Still, 12minute 30 second loop, H.D. Video, projected.

**Figure 9.**
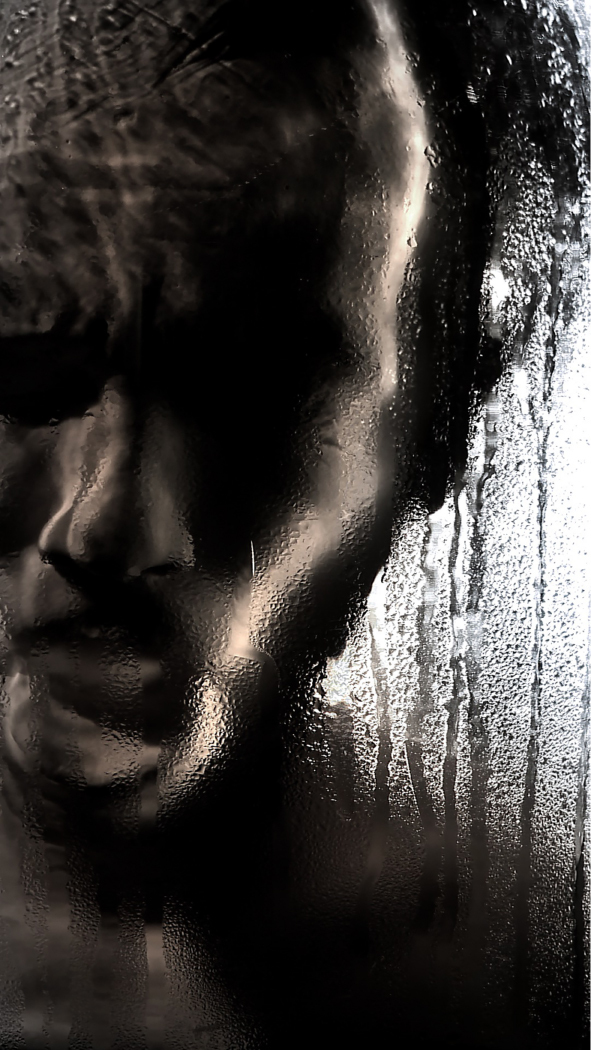
Christine Borland, NoBodies, (Detail) Choking Charlie, 2009, Film Still, 10 minute loop, monitor.

### Christine Borland interview with Craig Richardson, Glasgow Sculpture Studios, 19 November 2010

Borland: This plaster cast is from a fibreglass mould taken from an original plaster cast. The original was lost; the fibreglass started me on the quest to find the original, which I found eventually. I tracked it down from hearsay. It had been spotted in a museum store in the Anatomy building of Edinburgh Medical School with its extremities broken off. Looking at it now, I’m not going to put it back together permanently but to make the new cast I’ll make a partial repair, otherwise it's a significant conservation job. The Edinburgh University Anatomical Museum used to be over a number of floors of the Medical building. Two magnificent Asian elephant skeletons remain placed at the entrance. Through the years the Anatomical Museum was squeezed to make more space for teaching and by the 1980s many things were taken off display and hidden away in vaults, for example, there is a huge phrenology collection including skulls of indigenous peoples, some of which have since been repatriated. There is an attic space, which still has William Burke's (of Burke and Hare) skeleton and a life mask of William Hare. But the store is not a systematic academic presentation, there are random artefacts everywhere, crammed into a basement in a way that can’t help but resemble a gothic horror. It's certainly not set up as a teaching resource, unlike the Hunterian Museums in Glasgow or London.Richardson: With nothing catalogued and no proper documentation, how did you set about finding this lost plaster cast?

B: Via the curator of the Anatomical Museum. Dr Gordon Findlater, Director of the Anatomy Department at Edinburgh, is a proper enthusiast and is completely distraught that all these damaged things often lack of any proper documentation. It was in a corridor alongside other neglected and damaged objects.

R: Assuming this is then a relatively recent plaster cast does it have a named maker? Similarly had the more contemporary fibreglass,^5^ or even the original plaster if the one you located is not the true original?

B: Yes, and no. The fibreglass cast of the same figure in the centre of the RCS Edinburgh is labelled ‘a cast from nature, 1845, by John Goodsir’. Goodsir is credited with post-Burke and Hare restoration of anatomy's reputation in Edinburgh following the 1832 Anatomy Act. He was also renowned for his interest in art, music and for his humanity. But still there are no details on anything to do with an artist or at least there's no proper documentation as far as I know. Of the figure the Medical Historian Ruth Richardson has suggested that, although the dates don’t match exactly, that the figure could be a cast of a dissection mentioned in Goodsir's memoirs ‘an Edinburgh carter of intensely whisky habits, who in a drunken state fell from his cart and died on the spot [and] remained free from decomposition during thirty days.’ (2011, 2073–2074)

R: Let's talk about the recovered cast and the bio-information you notice.

B: When you consider the upper chest, neck – are these folds of skin or drapes of cloth? The cloth around the head could cover a major head injury I suppose, supporting Ruth Richardson's idea. It certainly seems to originate from a direct cast of a dissection. This is cloth draped over the groin area, I think by the way it is cracked that it is an actual piece of cloth, rather than something that has been moulded. And looking at the feet (it was easier to see when they were still broken off the main plaster section and detached) because of my little forensic knowledge I know that although they are now suspended in repose, that after death they were resting on a hard surface and that is commensurate with a conventional dissection with the back of the soles resting on the table. In other words evidence of resultant hypostasis and the indelible imprint of what they were resting on. Therefore the posing must have happened post-dissection, possibly in preparation for casting.

**Figure 10.**
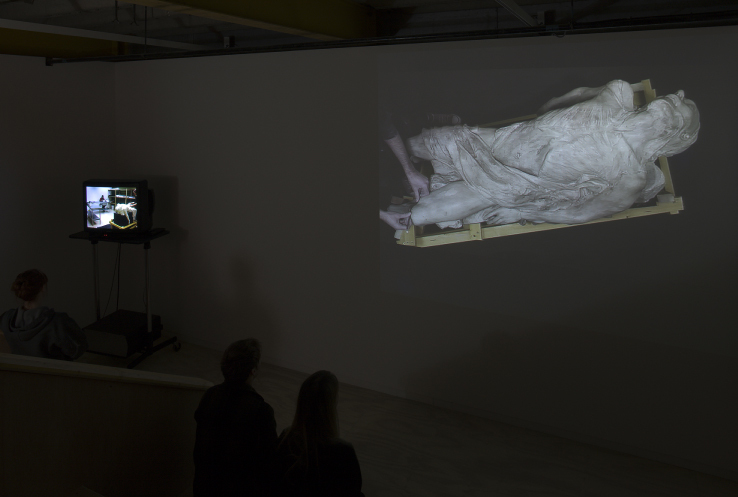
Christine Borland, Cast From Nature, Glasgow Sculpture Studios, 2010. (Photo: Ruth Clark)

R: Looking for further evidence of post-dissection intervention, perhaps towards the creation of an artwork, would the person posing the body have followed instructions?

B: The usefulness of this cast as a teaching tool would always have been doubtful, in fact the detail in the dissected parts is not that good and frankly how useful is *that* type of modesty (pointing at the figure cast section of draped cloth) in a medical context? The pose is in a distinct classical tradition, Michelangelo's Pieta, so its sculptural qualities matter more than its medical education potential.As the posing follows classical traditions there is an obvious comparison with the recent project by Joan Smith, who teaches anatomical drawing at Edinburgh College of Art, who worked with an anthropologist Jeanne Cannizzo to study a decrepit plaster cast ‘écorché’ (trans: a flayed figure) in the Edinburgh College collection (and since badly stored in a cupboard) which is posed after the *Dying Gaul* (unknown artist). That cast's nickname ‘Smugglerius’, in mock Latin, noted the criminal past and the classical pose of the originating figure. Smith and Cannizzo found out that it was likely to be James Langar, a convicted highway robber, taken from London’s Newgate prison, hanged at Tyburn in 1776 and then dissected the next day by William Hunter (1718–1783), of the brothers John and William Hunter, both famous anatomists. William Hunter moved back to Glasgow, ergo the Hunterian Collection.

**Figure 11.**
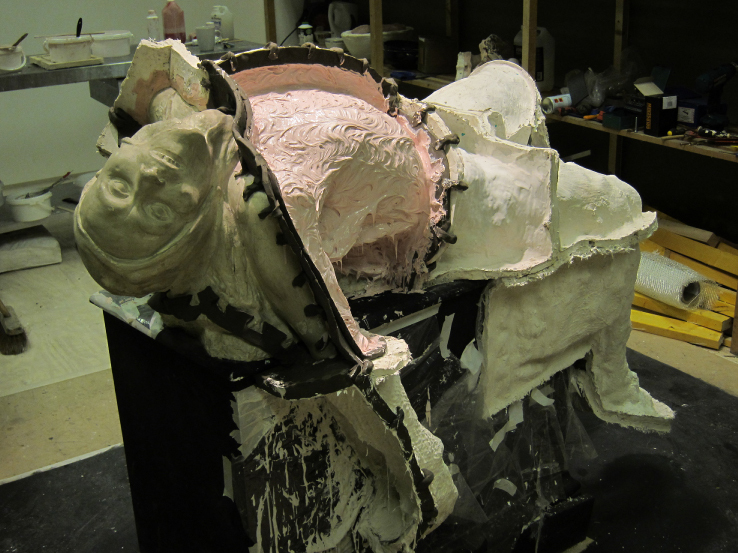
Christine Borland, Cast From Nature, Glasgow Sculpture Studios, 2010, Casting in Progress.

R: And have you tried to find out who made the original cast of this Edinburgh carter, or this plaster copy for that matter, or does that matter?

B: I’ve tried to find out if the information is readily available, I’ve looked through available records and have asked conservators and curators, nobody in the RCS is able to tell me. The fibreglass cast of this, which is on permanent display, is credited to Goodsir and a particular date but then Goodsir's life predates fibreglass, so the plaque may refer to the plaster original. But then there's a little cardboard label saying this fibreglass cast of the object was made possible by a grant from the Museum of Scotland, so I went to them to ask if they had records of the grant and they had the name of a sculptor who had made the fibreglass. Originally I was ambitious enough to ask ‘do you have the moulds’ that must have been made to produce the fibreglass cast.

R: Okay. So would this kind of casting have any medical purpose?

B: Well that's interesting, what I was describing there was a fibreglass cast that was made in the 1986. Nobody could tell me why it was made. Perhaps the original plaster was in a poor condition. There must have been a plaster original that the fibreglass cast was made from but the one I’m using here isn’t it because when you make a fibreglass you always leave traces on the original, so this definitely isn’t the one that was used because of the lack of fibreglass on any of its surfaces. This is not the source of the 1986 fibreglass on display. Incidentally, with fibreglass casting the more casts you make from the original the worse quality it is. The figure in the RCS is shrunken in scale because every time you cast you take away some of the surface, really a large percentage from the overall area. The next generation of cast is 12% smaller. But as for any medical purpose, I can only imagine the making of the original plaster cast was for artistic reasons. I don’t know if an artist or technician would have made it, you would need some sort of historical knowledge of the material that I don’t have. Gelatine might have been used for the inner mould.

When I make a cast of this the substance I will apply is a silicone rubber, a rubber mould captures all the detail, then it is wrapped in a jacket of plaster or some such material as a rigid case for the rubber that is doing the work, then the plaster will be poured in to the rubber mould. When the original cast was made of the dissected body, rubber was not available in 1845 so I can only speculate that plaster was *directly* applied and that's a horrible image to me because I know what you have to do to physically remove it.

R: So the process then had a salacious aspect, even if used here for more elevated purposes?

B: The overall pose suggests the decision was taken to not just to cast the dissected area as a record, there is no dissection of the legs and arms and relatively little of the face; the actual proportion of the body that is dissected is relatively small.

R: Thinking of other instances of artists attending dissections and invited to make something visual, perhaps this posing and original cast is such an example? In the absence of relevant historical information, your gut instinct will provide us with the best speculative insight. So, speculatively speaking, the posing of the body to resemble a Pieta, is this an artistic intervention?

B: No, not in the sense we mean it now. Even if an artist had made the cast I would say they would be acting under instruction of the anatomist, I very much doubt the artist would be making the decision about the pose, I would really feel the artist in this case – if indeed it was an artist or more likely a technician – if the artist was asked and commissioned to make the pose, they would have been asked to make use of many references, in this case referring to the Pieta. From other things I have looked at in the period the ‘voice’ of the artist or an independent aesthetic decision-making of an artist really is not discussed – the person who is taking those decisions is the anatomist, such as Hunter. It is then credited as the work of the anatomist, no artist mentioned there and any aesthetic decision-making is an expression of the medical professional.

R: So accepting some of your points about an obscure purpose in an anatomical-training setting, why make the original cast?

B: As a memento of the skill of the anatomist? Which is rather dangerous territory. Or to mark the anatomist's belief the elevation of the status of the body.

R: Could the cast have consciously been made as an example of technical display rather than artistry?

B: It seems unlikely, though that might have been a factor if there was a destination in mind for the cast – maybe as a centre piece for a museum, as it is today in RCS Surgeon's Hall. It could have been for teaching purposes, that *is* possible, but who were the students? Sculpture Students from the Art School? There certainly doesn’t seem to be any record of the artists/sculptors or technicians involved. At the same time, I just feel that, speaking with anatomists, there is little practical learning to be gained from this cast; it is too removed from a scientific context. This cast is a kind of elevation, away from the obvious ethics associated with this science, the immediate reference being more to a Pieta – like a Dead Christ figure.

R: Would an anatomist at this time be engaged with something other than seeking to understand bodily functions?

B: These contradictions are built into anatomical dissection, what was happening was actually quite horrific. Before 1832 the bodies came directly from the gallows and dissection was a public spectacle. In the earliest Anatomy School Fabricus, Vesalius (1514–1564) introduced elements to the anatomy theatre like engravings and inscriptions such as ‘The dead teach the living’. That kind of attempt to elevate through art includes the early anatomical texts accompanying the flayed dissected bodies, posed with various references to classical art, in Albinus, Vesalius, etc., but also building towards a representation of an interior mapping of the body.

R: In other recent works you’ve been looking at the technological modern version of simulated patients. You consider these do not encourage an empathic response, and the medical students have tended to treat the simulated figures as caricatures. But there seems to be, in this earlier cast in front of us, an attempt by the profession to encourage an empathic response. Do you think that is actually more conducive to the encouragement of empathy than the how you’ve witnessed the application of modern technology?

B: Yes, is the short answer. It's what I like to explore in discussions with contemporary medical and anatomy students and teachers. I suppose this cast directs me to wonder whether the actual physical process of dissection can be explored further through an emotional context, a context often completely absent from new teaching methods using contemporary simulated patients. As far as I can tell, in the world of anatomy education the students often experience extreme emotional reactions to dissection, which are not explored or even considered a valuable learning experience. Sometimes students are told to just put on a brave face – just get on with it! Teenagers confronting a dead body for the first time, those anonymous bodies donated to science. But by taking away this emotional engagement and replacing this with over-dependence on mannequins and simulated patients, a lot seems to be getting lost, ultimately a loss for future patients.Glasgow University still values anatomical dissection, their students work with both plastinated specimens (the procedure patented by Gunther von Hagens) and preserved cadavers. I’ve been in and out of the labs there for more than 20 years. A really gifted teacher and anatomist Dr John Shaw-Dunn, and Dr Quentin Fogg, have been exploring how the Humanities and Fine Art in particular can be brought into day-to-day teaching. Fogg put me on to a documentary made in a medical school in New Zealand, with students dissecting the same cadaver over a period of two years. The students will strip it down and empty it out over the course of the years. It is very, very intense. The medical students are followed on the journey and their emotions are explored, how they form a narrative of the person and their life. We also see them as they attend the memorial service for the donor and meet the families. A prior recording was made in which those who were planning to donate their bodies, the bodies the students have dissected, were interviewed about their motivation and their decision. Some were terminally ill patients or simply old and they nicely described what they thought of their own body, they said things like ‘I think my heart is really strong’, ‘my lungs will be shot with the smoking’. Obviously they had been prompted to think of themselves, as they will be shortly, as cadavers. After two years, at the conclusion of the longer-term dissection and having learned a great deal, with some forming their own narratives of the dissected person from their own personal perspective, the students were given an opportunity to view the recorded interview with the donors. Some of them took up the opportunity.

R: In earlier works you’ve been through a similar process, having gone through the ephemera of a forensically recovered body, but declined to include the background, contact, relationships or biographical information.

B: I made a decision to stop, it is enough to point out the context includes an historical context but not to bring it down to the nuts and bolts of an investigation. Viewers can fill it in for themselves. It's the opposite of what those Medical students did when they chose to watch the film interviews of the body donors. That level of detail isn’t helpful for an artwork.

R: I suppose we are talking here about posthumous but visual genealogies again, the visual information gained from the dead, but then let's say this is my ancestor's cast here, I’d be keen to find out, from a genealogical fascination.

B: you could do that, but that's not *my* way! I’m bringing this into the public realm, I’m taking a cast of someone which hasn’t been looked at for decades, who has been climbed all over, and broken, and now brought out of the basement. If anyone wants to do that type of genealogical research I would be really delighted, but I don’t want to do that. What I found interesting about the New Zealand medical students were not those who choose to hear the stories but those who choose *not to* hear the stories on the prior recording, they had found a means to come to terms with the experience of the donated body through their own engagement and by their own stories. That's what I found most interesting, the comparison of the students’ narratives with the real narratives. These students managed to find a way to come to terms with it in a really positive way by being given a forum to talk about feelings that didn’t ‘fit’ in a medical context. When they had to remove the heart, which in scientific terms is just another piece of the puzzle of the body, to many of them that had a symbolic significance and meaning, which in this medical context was difficult to talk about.

R: Do they talk like that? Do they talk from that ethical position?

B: Yes, absolutely. I don’t know if it's that's the purpose or a by-product of this discursive film-making process in New Zealand but that's what happens. The students value it and speak in terms of it having been the most important two years of their lives. The ones planning to become surgeons, who might end up spending a lot of time operating on people who come into Accident and Emergency wards, they’ll never meet them until they’re on the operating table. They talked about how this experience would help with that moment, having had such an intimate engagement and over those two years to build that ethical code for themselves from their own perspective.

R: This all seems far removed from this idealistic Pieta cast in front of us now, this cast doesn’t seem then to invite opening up any exploratory biographical channel to the students.

B: No, it has been completely removed from any sense of a day-to-day, nineteenth century existence. It might be doing that top-line job that you sort of introduced earlier on, ‘isn’t anatomy crossing the line, towards an artistic form’. Hunter and other anatomists think of themselves as artists and performers, ‘it's an artform’ that's said all the time. So it's a form of expression of that …

R: do *you* think it's an artform?

B: Emm, maybe unfair to ask me, I’m too involved at the moment. There's been lots of peripheral research that has helped me think about it and make decisions. The reference to Pieta … the only one I could have direct access to is one in the Edinburgh College of Art cast collection, only the dead ‘Christ’ now has the figure of Mary gone, excised. You are left with a figure that looks a lot like this cast here. There are still lots of folds of drapery, which we know from the original is Mary's skirt. If you look carefully, where this phony rock is, there is a hand but no Mary. So the support is obvious, if you know what you are looking for, under the voluminous drapery. But She's absent. But I had a feeling of *that* whenever I saw this cast, I thought about the suggested support right away, the physical bonding. It's been mocked up here, the roughly emulated rocks which hold him in position now. But someone, maybe many people, would have lifted this guy, placed him in position to cast. When I make the cast I won’t include such fake supports I’ll just have him free floating, and once I have the new cast I want to work with that, to make a new extended support structure, but no plinths or rocks.

R: When you devise a new support as an extension of the sculpture, what do you think you will create as a support?

B: There are many possibilities. One of the things I want to do with the new cast is use a technique resulting from an accidental part of the casting process – the films I made of the plaster cast heads shown in Belfast of simulated patients (the exhibition *SimBodies* & *NoBodies*). Once I made the plaster casts I took them out of their mould while they were steaming hot, I wasn’t sure where it was going at that point. When I put them under glass the condensation obscured them but over time it started to clear and their features became visible (Figure 9). I may do something like that; the cast will be put in a Museum case, it will create a big smokescreen obscuring the object, but the cast will gradually reappear. I’d like to try that.(*We move round to the underneath of the cast where the neck and base meet*) If you look inside here this is the hole which if you look inside with a torch you can see the fingerprints of the person who made the cast. This is the reverse side, all their finger marks of how they pushed the plaster into the mould is there. I was amazed when I saw the Edinburgh Pieta because you can get round to look at it from the back. It's propped up with a series of iron rods which you’d expect but when you look closely they are just branches, just stuck in. It has an incredible violence and traces of hand-marks particularly when you consider it's the space behind Mary's legs and that's the hand of the sculptor.

When I do this cast what we have on the outside will be a replica of this sculpture but inside will record my hands and my touch. I’m not replicating the existing support so when you look up from underneath it will be hollow, you can clearly see this evidence of my hand.

R: Returning to the contemporary mannequins, ‘Choking Charlie’ and so on, is there any linkage in thinking about the inside of this plaster cast?

B: Only some of the mannequins are cast from life, transformed into flesh-tone latex and rubber, then bits of mechanics and computing technology are inserted. There is a rather lovely and widely used mannequin who is a living Japanese basketball player, he is a heart-monitor simulation figure, his figure lies on a table and is modelled only to the top of his legs. But most are composite figures, an artist must have modelled them at some point in time but the seven I chose to work with in my exhibition in Belfast (*SimBodies* & *NoBodies*) were a mixture of obviously lifelike starting point and then obvious composites. Of more importance in a training context are the internal organs; the way the models look, their face, has no real medical educational benefit (Figure 8).

R: Not portraiture then, but ‘faces’. A lot of which appear in many of your previous works. The face in this cast has a highly emotional contemporary content, it is redolent of images associated with the twentieth century history, famine, war, Holocaust.

B: That's important – the historical anchoring. But of course it just feels familiar from that kind of imagery, we don’t need to be explicit about it.

R: In the Glasgow Exhibition there will be a separate viewing area with a live film feed, showing the cast as it is underway (Figure 10). In the same way you are fascinated by some of the techniques you see or hear about in anatomy, art processes and their demystification might fascinate others?

B: I feel a certain relief as well and it's been built into the decision-making that I’m not actually physically representing the spectacle of the sculpture as part of the show, in its raw, broken and undignified form – there's plenty of that conveyed in this mediated image, providing me with a bit of relief that I’m not further exploiting the actual physical being. We talked about it before, he artist's rules, one's own ethical code, and this fitted with mine, not to have the original object physically present.

R: On the theme of the exploitative, there has been a live dissection broadcast on television, which you might correlate to historical live dissection in an actual space as it were, perhaps this was regulated event which might have been more dignified, perhaps it's inevitable that the television image would bring into question the dignity of the event. This new space you’ve made, its low lighting and tiered seating, it brings a certain dignity to the event?

B: There's a myriad of references but essentially it's an intimate experiential space which hopefully can be valuable on a one-to-one basis – and we’ve talked about the experience of seeing *that* kind of pose and *that* kind of expression [of the sculpture] mediated through news broadcast media – but by making this very particular space to experience this work, I hope I can enable other possible forms of engagement. Yes, you are viewing a projected image on a screen the same as all that news footage but the physical context is so very different, as a viewer you are allowed to have different thoughts and so on.

R: The risk here would be that this is described as a sculpture being transformed into a single fixed image whereas this viewing room and the second projected image showing the place of construction is about the spatial experience. It is redolent of other types of viewing experience in history and the contemporary realm. Incidentally, thinking back to next door and looking at it onscreen now, your casting room looks explicitly medical or is that my imagination?

B: These tables were borrowed from the casting workshop next door and all these pots are the real thing. When I showed the staff here in Glasgow Sculpture Studios images from Pathology laboratories they immediately noticed exactly how like this casting set-up it looked, full of plastic pots, cutting and scraping, measuring and stirring things.

## Notes

1. A fascinating advocacy for such performed interaction is expressed in Kohn's ‘Performing medicine: the role of theatre in medical education’: Theatre in particular can provide irreducible experiences emanating from the realms of flow knowing and whole knowing. Flow knowing is knowing in relation to others and to our environment. It is expressed through narrative and kinesthetics – our stories and bodies in spatiotemporal communication with our fellow creatures and the world. Whole knowing comes in a flash (epiphanically) through engagement with the arts, and panoramically, through engagement with synoptic disciplines such as history, philosophy and religious studies. The combination of flow and whole knowing, with empirical knowing, grounds every medical story within the context of a human story. It also moves our students to meld their ‘being’ (values, character and beliefs) with their ‘doing’ (technique) towards creation of a full and reflective practice life. *Medical Humanities* (37): 3–4.

2. Related works appeared in Niet Normaal: Difference on Display (Bluecoat Gallery, Liverpool 21st Jun-9 September 2012); also in the exhibition *Cast Contemporaries: artists respond to the completion of the Cast Collection Project* (Edinburgh College of Art, 2 August–2 September 2012, curated by Chris Dorsett and Margaret Stewart); see http://castcontemporaries.weebly.com

3. The cast presents an interesting avenue of research for students of the history of Scottish photography. The Scottish anatomist John Goodsir studied Arts at St Andrews University. In 1840 Goodsir went to Edinburgh and in 1841 he was appointed Conservator of the RCS Museum, and in 1842 he moved to the University becoming Curator of the University's Anatomical Museum. The original sculpture was photographed in 1847 two years after its creation and the archives at Surgeons Hall Museum include a unattributed ‘Copy of a wet plate photograph taken in 1847 which was in the possession of Dr William Guy. Presented by D. J. Menzies Campbell’. This is probably Dr William Guy (1810–1885). The linkage of surgery to photography at this time is found in Guy's contemporary Thomas Keith who, with his brother George Skene Keith, both were members of Sir James Young Simpson's team that pioneered the use of chloroform as an anaesthetic. The Keith's were early proponents of photography with George as a founder member of the Photographic Society of Scotland, and who first exhibited his work in 1854. The Keith's father Rev. Dr Alexander Keith sat for calotypes taken by Hill and Adamson around 1843.

4. For a fascinating response to the USA’ growing aversion to the once-hallowed plaster cast as imitation and how ‘dutiful study’ was supplanted by the ‘aesthetic uplift’ of temporary exhibitions within collections such as the Metropolitan Museum see Siegel, K. 2011. p. 140: ‘However plaster casting continues unabated in contemporary American art, George Segal, Jasper Johns, Allan McCollum, and the art of the reproducible and of a simulacra is one of the pillars of contemporary Western art’. Siegel continues her discussion of plaster casts on p. 147.

5. In correspondence Andrew Connell at the Surgeons Hall Museum posited an idea that Arthur Wyllie, conservator of the National Museums of Scotland, and his assistant Brian Melville, may have been responsible for the fibreglass model now on permanent display. Wyllie was a graduate of Edinburgh College of Art and a contemporary of the Scottish artist Joan Eardley.
